# Primary Oesophageal Lymphoma: A Diagnostic Dilemma

**DOI:** 10.7759/cureus.73751

**Published:** 2024-11-15

**Authors:** Varun Sidana, Rohan LNU, Anna Davenport, Venkata Lekharaju

**Affiliations:** 1 Internal Medicine, Wythenshawe Hospital, Manchester, GBR; 2 Trauma and Orthopaedics, Wythenshawe Hospital, Manchester, GBR; 3 Histopathology, Manchester University NHS Foundation Trust, Manchester, GBR; 4 Gastroenterology, Wythenshawe Hospital, Manchester, GBR

**Keywords:** dawsons criteria, diagnostic and therapeutic challenge, non-hodkins lymphoma, primary oesophageal lymphoma, rare cancer

## Abstract

Primary oesophageal lymphoma is an exceptionally rare cancer originating in the esophagus, distinct from more common oesophageal malignancies. Dawson's criteria has been the mainstay for diagnosis for such entities. However, recognizing primary oesophageal lymphoma is particularly challenging due to its rarity, diverse clinical presentations, and non-specific radiological and endoscopic features. These factors often lead to diagnostic delays, complicating timely treatment and resulting in poor patient outcomes. We hereby present a challenging case and literature review about this rare malignancy.

A 78-year-old male was referred to Gastroenterology for evaluation of dysphagia. Initial esophagogastroduodenoscopy (OGD) revealed an oesophageal ulcer with features consistent with oesophageal candidiasis. A follow-up OGD identified an oesophageal stricture. Histopathology and immunohistochemistry of biopsy samples confirmed a diagnosis of diffuse large B-cell lymphoma (DLBCL). A staging CT was completed, and an oesophageal mass was shown extending into the mediastinum. The patient re-presented with worsening dysphagia, renal dysfunction, and hypercalcemia. Although lymphoma was confirmed, to ensure an appropriate regimen is commenced, a repeat OGD was recommended by the Hematology team. The patient's rapid deterioration necessitated pre-phase steroid treatment and management of tumor lysis syndrome. Before R-CHOP chemotherapy could begin, the patient deteriorated further. Following a multidisciplinary team (MDT) meeting and discussions with the patient and family, it was decided to manage the symptoms with palliative intent.

This case highlights the diagnostic challenges posed by primary oesophageal lymphoma, emphasizing the importance of considering this rare malignancy in the differential diagnosis of oesophageal strictures, particularly in elderly patients. Aggressive disease progression complicates managing DLBCL in older patients. Moreover, this patient population has other co-morbidities that would preclude treatment options at the outset. Further studies are needed to establish optimal diagnostic and therapeutic strategies for this rare condition.

## Introduction

Lymphomas represent a diverse group of malignancies originating from the clonal proliferation of B-cells, T-cells, and natural killer (NK) cells at various stages of maturation, accounting for approximately 5% of all cancers [1]. These malignancies are broadly categorized into non-Hodgkin lymphoma (90%) and Hodgkin lymphoma (10%) [2]. The presence of Hodgkin and Reed/Sternberg (HRS) cells is the defining feature of Hodgkin lymphoma [3]. In contrast, non-Hodgkin lymphomas (NHL) are a varied group of lymphoproliferative malignancies that are less predictable than Hodgkin lymphomas [4]. NHL can be further divided into 'indolent' and 'aggressive' subtypes based on disease prognosis [5].

Primary extranodal lymphomas are an uncommon presentation of non-Hodgkin lymphoma, characterized by minimal or no nodal involvement and a clinically dominant extra nodal component that often dictates the treatment approach [6]. The gastrointestinal (GI) tract is the most frequently involved extranodal site in non-Hodgkin lymphoma [7]. However, oesophageal lymphoma is particularly rare, comprising less than 1% of gastrointestinal lymphomas; these can arise from metastasis of cervical or mediastinal lymph nodes or the direct extension of a gastric lymphoma [8]. Primary oesophageal lymphoma in patients who do not have a history of immunological compromise is exceedingly rare [9]. Furthermore, the imaging characteristics of oesophageal lymphoma do not reveal any diagnostics specificity, complicating the process further [10].

In this report, we present a rare case of primary oesophageal lymphoma and highlight the significant diagnostic challenges associated with this uncommon condition.

## Case presentation

We present the case of a 78-year-old male who was referred to the Gastroenterology Department with a four-week history of dysphagia to both solids and liquids. The patient also reported fatigue, anorexia, constipation, and significant weight loss of approximately 19 kg over the preceding six weeks. His medical history was notable for hypertension, hypercholesterolemia, a previous myocardial infarction, and chronic obstructive pulmonary disease (COPD) with an exercise tolerance of less than 100 meters. His regular medications included simvastatin, nebivolol, clopidogrel, ramipril, and inhalers for COPD.

**Figure 1:**
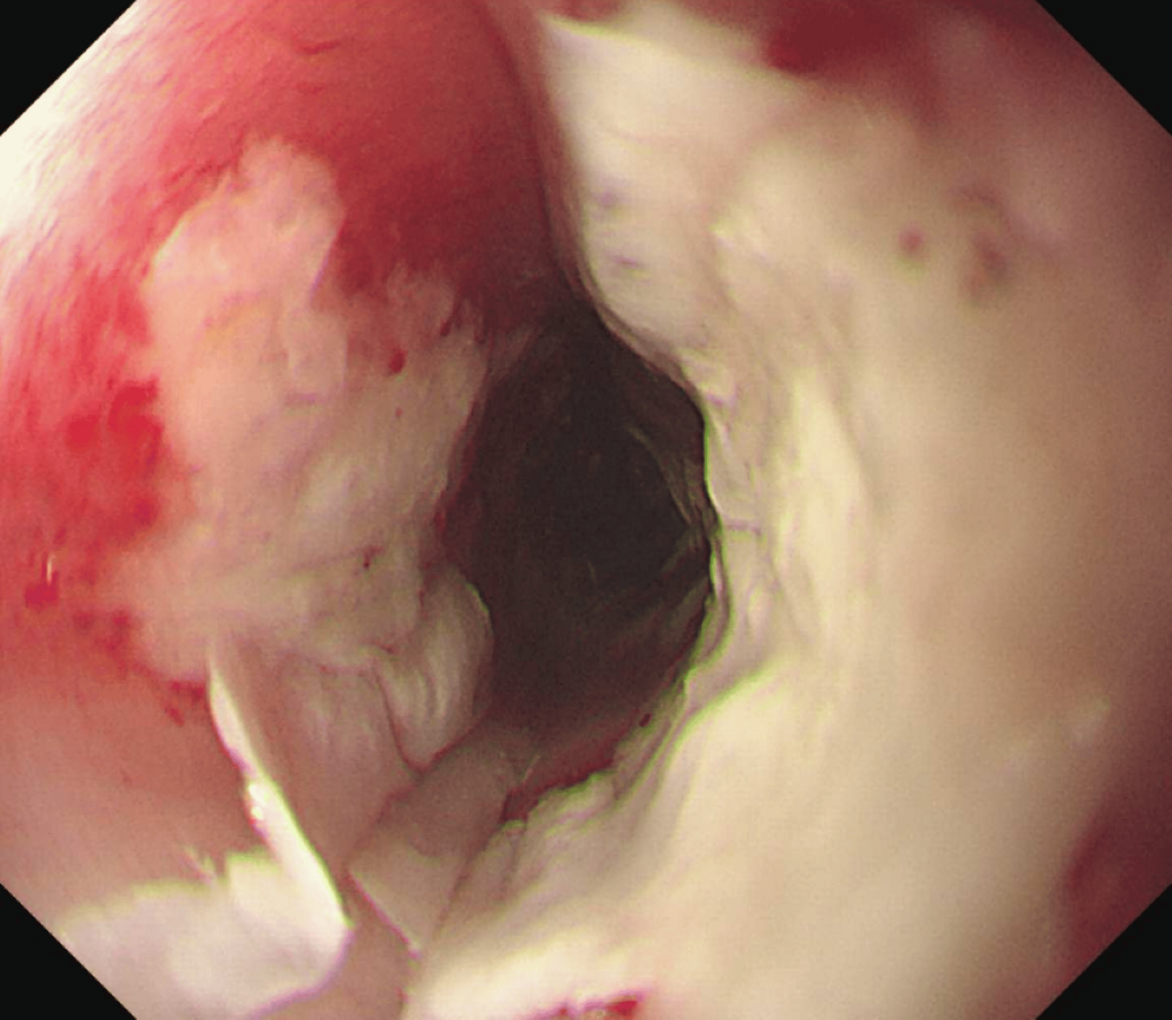
Initial esophagogastroduodenoscopy showing oesophagitis with extensive candidiasis

An initial outpatient esophagogastroduodenoscopy (OGD) revealed oesophagitis with extensive oesophageal candidiasis (Figure 1), and subsequent histology returned negative results for malignancy. The patient was discharged with a proton pump inhibitor and oral fluconazole, and a follow-up OGD is scheduled in two weeks to ensure the resolution of symptoms.

**Figure 2:**
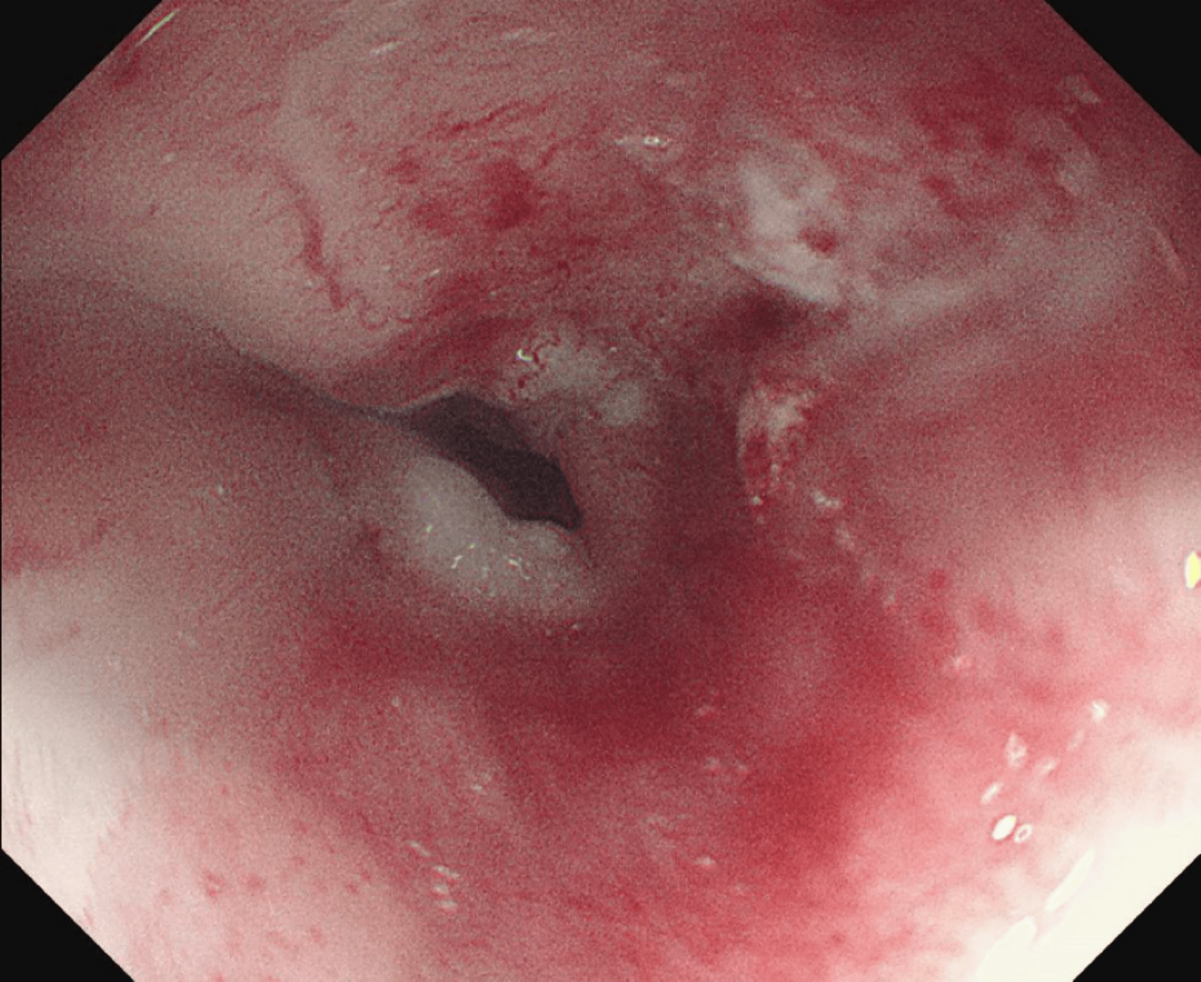
Repeat esophagogastroduodenoscopy showing stricture and narrowing in the oesophagus with neovascularization and mucosa changes not explained by extrinsic lymph node compression

At the repeat, OGD, a moderately tight stricture was identified at 28 cm (Figure 2), and the overlying mucosa did not appear to show signs of neovascularization. A cold forceps biopsy was performed to assess for dysplasia, and an urgent outpatient CT thorax, abdomen, and pelvis (CTTAP) was arranged to evaluate for suspected malignancy.

**Figure 3:**
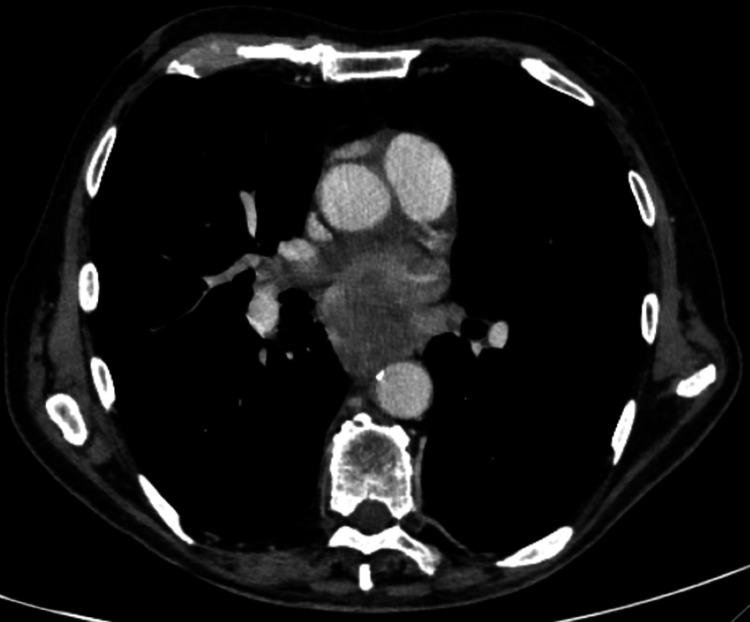
CTTAP revealing an ill-defined oesophageal mass encasing the left main bronchus CTTAP: CT thorax, abdomen, and pelvis

**Figure 4:**
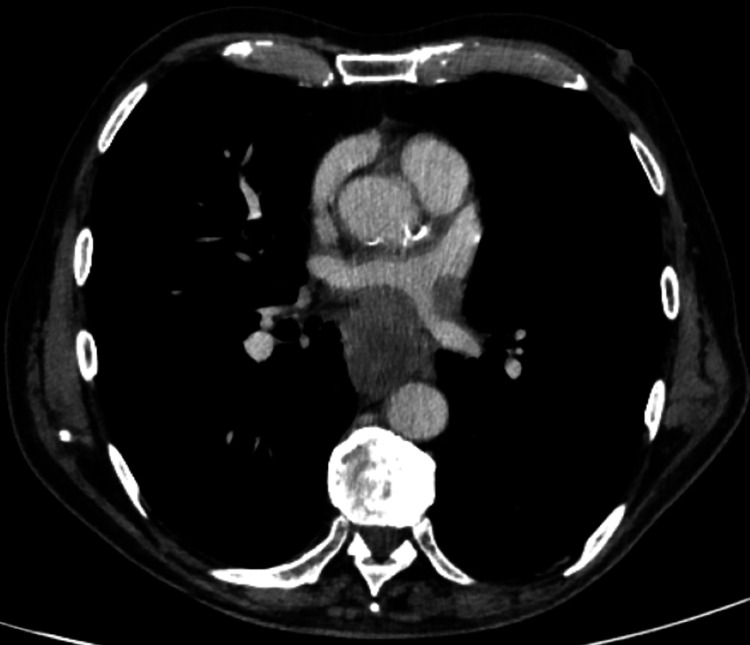
The CTTAP revealing an ill-defined oesophageal mass encasing the left pulmonary vein CTTAP: CT thorax, abdomen, and pelvis

The CTTAP revealed an ill-defined oesophageal mass extending into the mediastinum, encasing the left main bronchus (Figure 3) and left pulmonary vein (Figure 4). The mass measured 10.1 cm in its cranio-caudal dimension and caused significant oesophageal luminal narrowing with mild upstream fluid-filled dilatation, findings consistent with oesophageal malignancy. Additionally, there was notable lymphadenopathy, including a right supraclavicular node measuring 10 mm, a right hilar node measuring 30 mm, and enlarged mediastinal and right pre-tracheal nodes.

**Figure 5:**
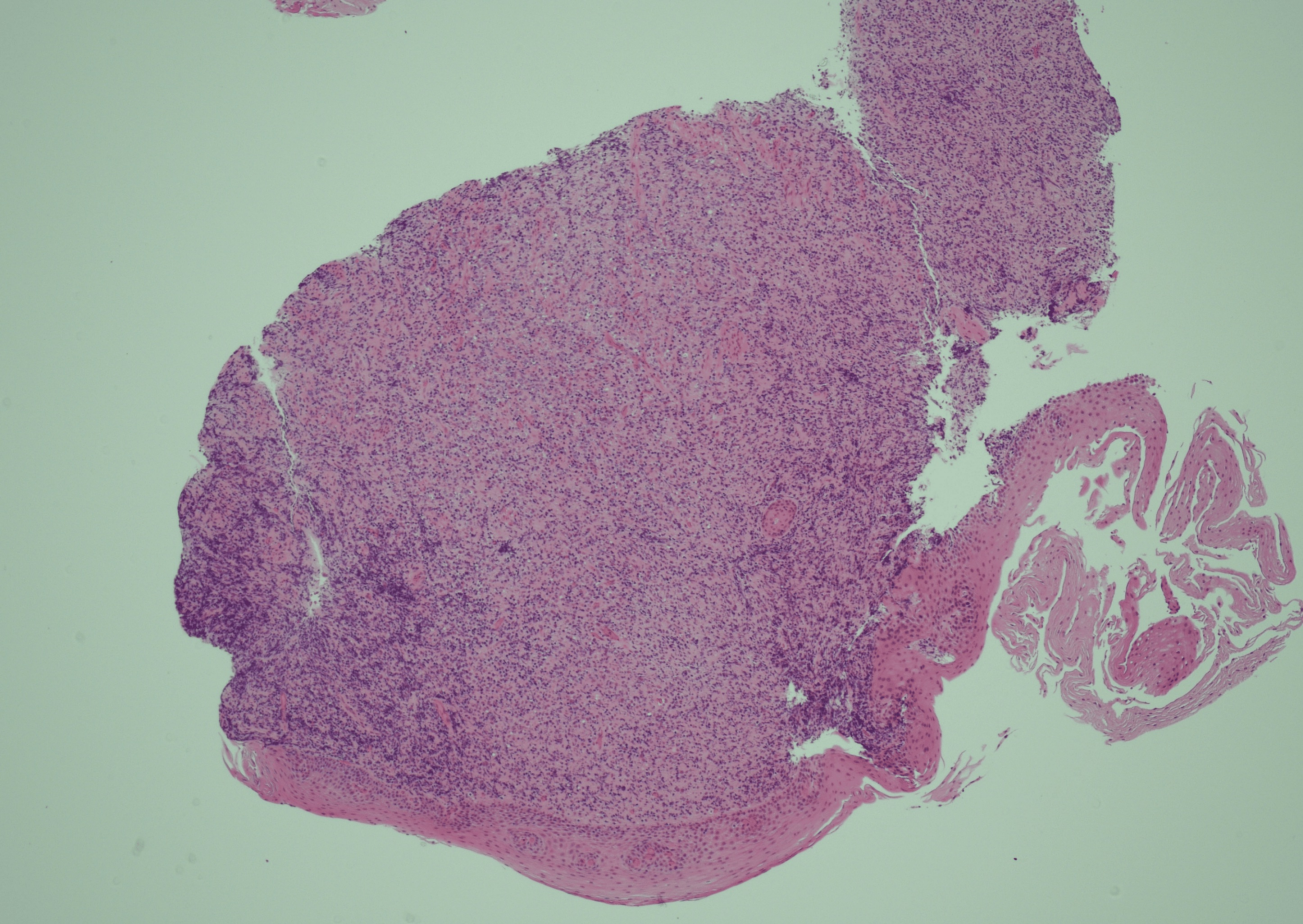
Squamous epithelium of oesophagus overlying cellular infiltrate of lymphocytes

**Figure 6:**
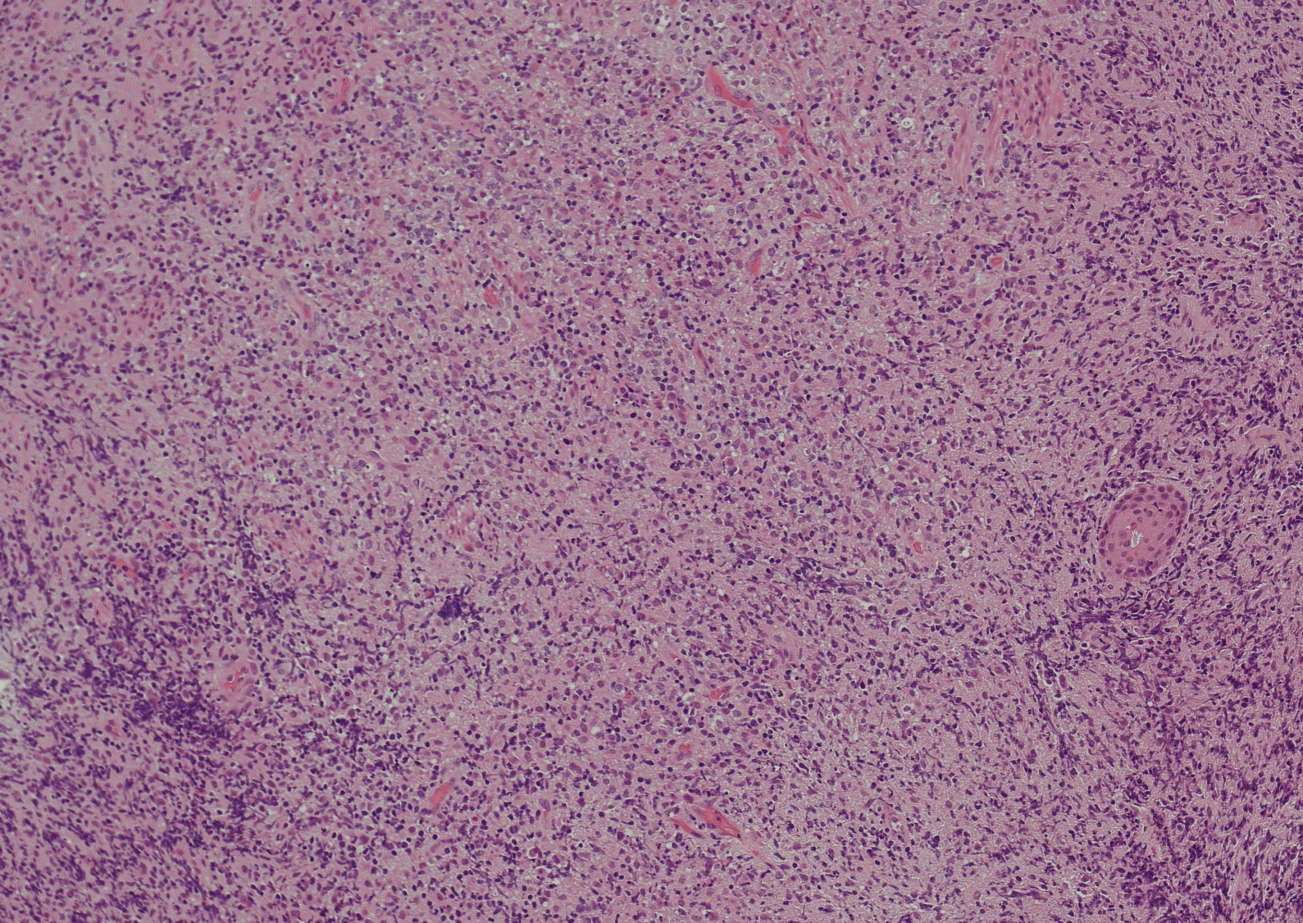
Close-up of lymphocytic infiltrate

**Figure 7:**
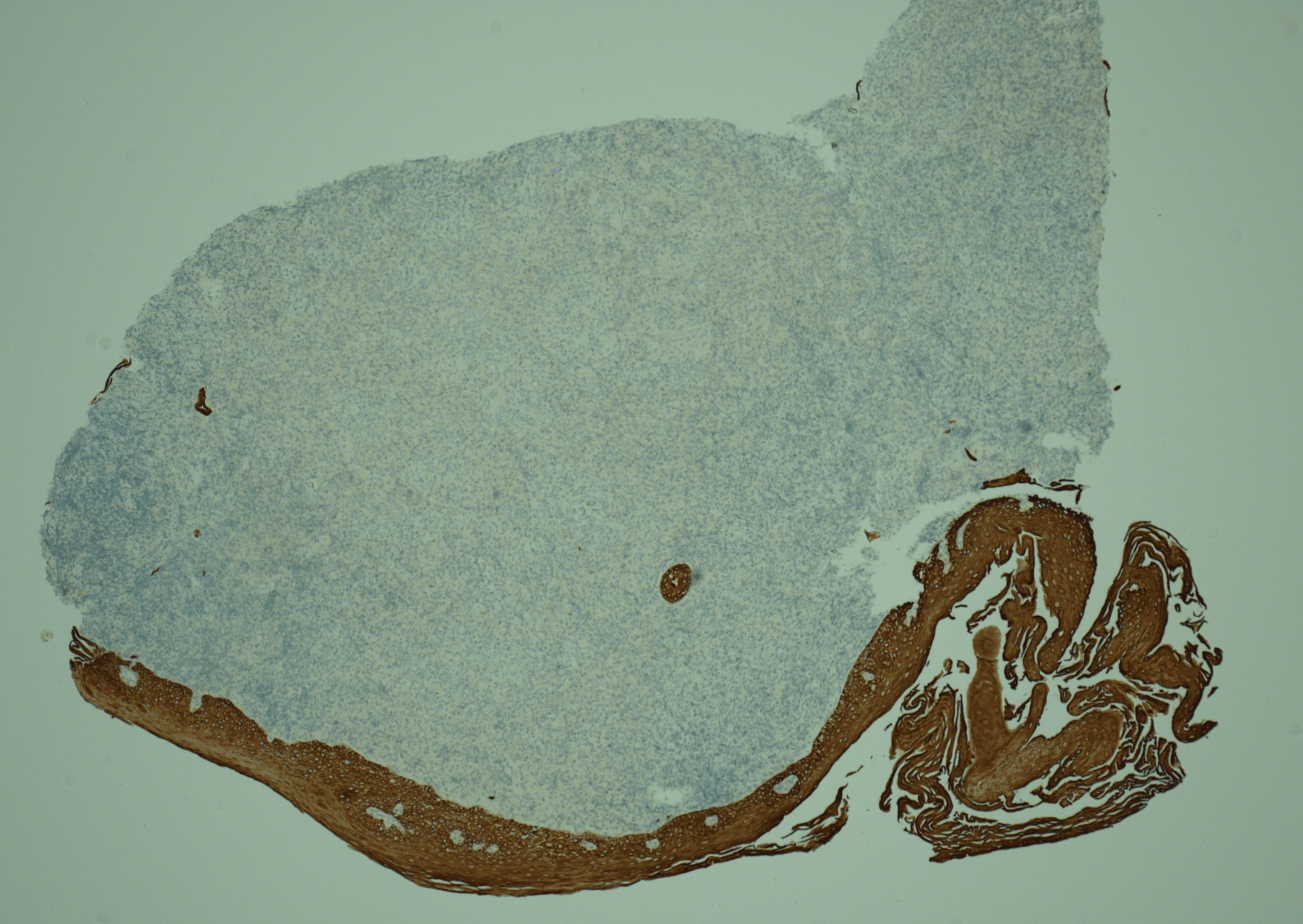
Cytokeratin stain (AE1/3) showing positivity in normal squamous epithelium but not in subepithelial cellular infiltrate

**Figure 8:**
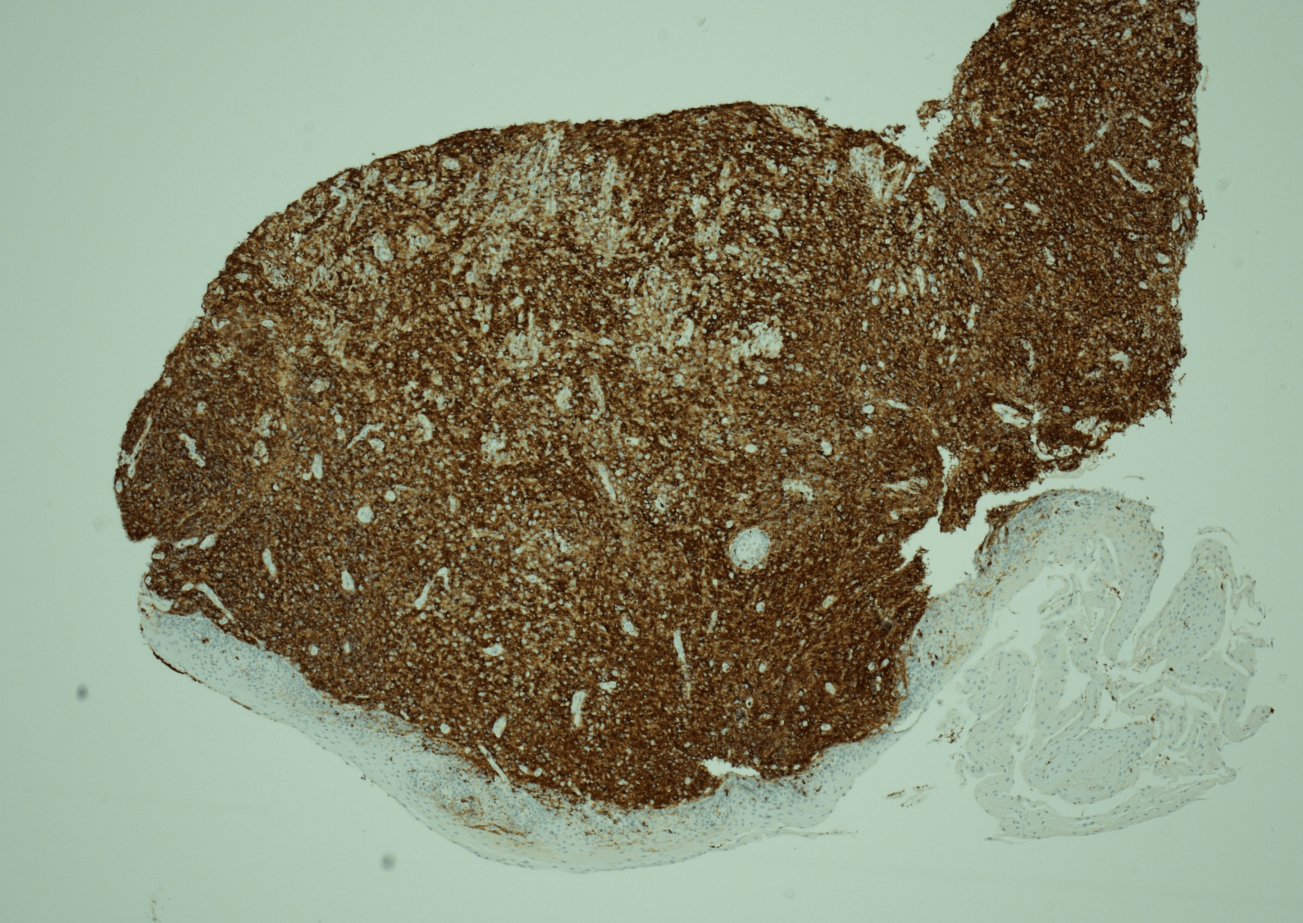
LCA stain showing that the infiltrate is lymphocytic in nature LCA: Leukocyte common antigen

The biopsy's histopathological analysis (Figure 5-8) revealed a dense infiltrate of cytologically atypical small to medium lymphocytes, with occasional larger lymphocytes and scattered apoptotic bodies. Immunohistochemistry demonstrated a prominent atypical B-cell population, positive for CD20, PAX5, CD79a, BCL2, MUM-1, and BCL-6, with weak CD30 expression. There was no evidence of BCL2 or BCL6 gene rearrangement in fluorescence in situ hybridization (FISH) studies. The overall findings were consistent with a diagnosis of oesophageal B-cell lymphoma.

Prior to the scheduled follow-up, the patient unfortunately presented to the Emergency Department with worsening dysphagia, acute renal dysfunction, and hypercalcemia. Physical examination at that time was unremarkable, with no organomegaly or lymphadenopathy. Initial blood work showed no anemia, a normal white cell count, and normal serum lactate dehydrogenase (LDH) levels.

An urgent inpatient referral to Haematology was made, and while lymphoma was the primary consideration, initiation of appropriate chemotherapy required further pathological subtyping. A right supraclavicular lymph node biopsy was also planned to establish the disease subtype. However, the patient deteriorated further, and a multidisciplinary team discussion, including critical care, hematology, and gastroenterology, with the patient's family took place. It was concluded that the patient should be managed with palliative intent. Sadly, the patient passed away shortly after.

Subsequent lymph node biopsy results received posthumously, demonstrated a patchy population of atypical large blastoid cells with features of Centro blasts within a background of smaller lymphoid cells, consistent with a high-grade, highly proliferative B-cell lymphoma of germinal center B-cell (GCB) phenotype. The blastoid cells expressed CD20, BCL6, BCL2, and CD30, while being negative for CD5, CD10, CD23, Cyclin D1, MUM-1, IgD, CD138, and EBER-ISH. The MIB-1 proliferation index was 70-80% within a background rich in reactive CD3+ and CD5+ T-lymphocytes.

## Discussion

Oesophageal lymphoma in itself as an entity is rare, and only a handful of cases have been reported worldwide. Dawson et al. [11] concluded that the diagnostic criteria of primary oesophageal lymphoma meant having the involvement of oesophagus, only local lymph node involvement, i.e., no peripheral and mediastinal lymph node involvement, unremarkable liver and spleen and normal granulocyte/normal cell count

The patient fulfilled the Dawson's criteria in terms of localisation of lesion, normal liver and spleen, normal granulocyte count but there was presence of mediastinal and peripheral lymphadenopathy.

Qu J et al. [12] analyzed and carried out a systemic analysis of clinical data of 15 cases of primary oesophageal lymphoma retrospectively to improve the diagnosis and treatment of this disease, highlighting the clinical, endoscopic, and pathological features. They discovered that 46.7% (7/15) of the patients presented with local lymph node enlargement (all patients, i.e., 15, had mediastinal lymph nodes, and two patients also had hilar lymph node enlargement ). Interestingly, one patient had distant lymph node involvement without local lymph nodes being involved. This concluded that the absence of mediastinal lymph nodes isn't necessary for diagnosing oesophageal lymphoma and requires additional study.

The most noticeable presentations of primary oesophageal lymphoma include difficulty swallowing (89%), loss of weight (67%), change in voice (33%), and epigastric pain (33%) [13]. These clinical presentations overlap with those of other oesophageal pathologies, with dysphagia and weight loss being the patient's primary complaints. This overlap contributes to the challenge of achieving a timely and accurate diagnosis.

Although imaging findings of oesophageal lymphoma are well described, they are often non-specific. They often consist of stricture formation, as seen in this case, or the presence of a mass within the wall termed an intramural mass, and intermittently polyp-like protrusion with or without ulceration, findings which would often point towards benign conditions [14]. On endoscopic assessment, the presence of an unmutilated mucosa, circumferential involvement of the oesophagus, and the hard and fibrous nature of the submucosal mass, the former two being present here, should suggest the possible diagnosis of lymphoma [15].

To complicate matters further, obtaining the ideal specimen for primary oesophageal lymphoma can be tricky as the area of involvement might often be located in the submucosa initially. The diagnostic rates can vary with endoscopy, percutaneous ultrasound, CT, and gastrointestinal barium meal, with studies suggesting numbers of only 88.1%, 52%, 67%, and 83% [16]. The presence of a negative biopsy on the first OGD, followed by failure of histological sub-typing on the second and eventually sub-typing in the late phase of the disease, presents clues towards difficulty in establishing the diagnosis.

DLBCL is often curable and carries a good prognosis with combination immunochemotherapy but becomes noticeably more challenging to cure as patients age and become frail. Old and frail patients with co-morbidities and poor functional status are considered the most difficult group for treatment. For the unfit and elderly, for whom an intent-to-cure regimen is not an option, less intensive palliative options are regarded as a more suitable approach [17].

## Conclusions

Primary oesophageal lymphoma, though rare, should be considered in immunocompetent patients presenting with non-specific symptoms and inconclusive radiological findings. There is ample evidence in the literature suggesting that this entity is often underdiagnosed or misdiagnosed. Obtaining an adequate biopsy specimen also remains challenging, as the lymphoma may predominantly reside in the submucosa, complicating standard diagnostic approaches. Repeated biopsies of the same lesion may increase diagnostic yield in such cases. Furthermore, transitioning from flexible endoscopic forceps biopsy to more invasive methods, such as thoracoscopy or mediastinoscopy, should be considered when initial biopsies are non-diagnostic, as they allow direct access to the lesion.

Additionally, refining Dawson's criteria, as suggested in existing literature, may improve the diagnosis and management of primary oesophageal lymphoma. The proposed diagnostic criteria, partially consistent with Dawson's original framework, include the following: 1) The lesion is primarily located in the esophagus; 2) Only local and regional lymph nodes, such as mediastinal nodes, are involved, with no peripheral or distant lymphadenopathy; 3) Absence of hepatosplenomegaly; 4) Mild to moderate enhancement of the lesion on CT imaging; 5) Normal tumor markers; and 6) Pathological confirmation of the disease. These criteria could offer a more structured and effective approach to identifying and managing this rare condition. 
